# A Focused Review of Smartphone Diet-Tracking Apps: Usability, Functionality, Coherence With Behavior Change Theory, and Comparative Validity of Nutrient Intake and Energy Estimates

**DOI:** 10.2196/mhealth.9232

**Published:** 2019-05-17

**Authors:** Giannina Ferrara, Jenna Kim, Shuhao Lin, Jenna Hua, Edmund Seto

**Affiliations:** 1 Global Burden of Disease, Institute for Health Metrics and Evaluation University of Washington Seattle, WA United States; 2 Paul G Allen School of Computer Science & Engineering University of Washington Seattle, WA United States; 3 Department of Kinesiology and Nutrition University of Illinois, Chicago Chicago, IL United States; 4 Department of Medicine Stanford Prevention Research Center Stanford University Stanford, CA United States; 5 Department of Environmental & Occupational Health Sciences University of Washington Seattle, WA United States

**Keywords:** diet, nutrition assessment, behavior and behavior mechanisms

## Abstract

**Background:**

Smartphone diet-tracking apps may help individuals lose weight, manage chronic conditions, and understand dietary patterns; however, the usabilities and functionalities of these apps have not been well studied.

**Objective:**

The aim of this study was to review the usability of current iPhone operating system (iOS) and Android diet-tracking apps, the degree to which app features align with behavior change constructs, and to assess variations between apps in nutrient coding.

**Methods:**

The top 7 diet-tracking apps were identified from the iOS iTunes and Android Play online stores, downloaded and used over a 2-week period. Each app was independently scored by researchers using the System Usability Scale (SUS), and features were compared with the domains in an integrated behavior change theory framework: the Theoretical Domains Framework. An estimated 3-day food diary was completed using each app, and food items were entered into the United States Department of Agriculture (USDA) Food Composition Databases to evaluate their differences in nutrient data against the USDA reference.

**Results:**

Of the apps that were reviewed, LifeSum had the highest average SUS score of 89.2, whereas MyDietCoach had the lowest SUS score of 46.7. Some variations in features were noted between Android and iOS versions of the same apps, mainly for MyDietCoach, which affected the SUS score. App features varied considerably, yet all of the apps had features consistent with Beliefs about Capabilities and thus have the potential to promote self-efficacy by helping individuals track their diet and progress toward goals. None of the apps allowed for tracking of emotional factors that may be associated with diet patterns. The presence of behavior change domain features tended to be weakly correlated with greater usability, with *R*^2^ ranging from 0 to .396. The exception to this was features related to the Reinforcement domain, which were correlated with less usability. Comparing the apps with the USDA reference for a 3-day diet, the average differences were 1.4% for calories, 1.0% for carbohydrates, 10.4% for protein, and −6.5% for fat.

**Conclusions:**

Almost all reviewed diet-tracking apps scored well with respect to usability, used a variety of behavior change constructs, and accurately coded calories and carbohydrates, allowing them to play a potential role in dietary intervention studies.

## Introduction

### Background

A large number of apps focused on health and fitness have emerged on the smartphone market. In 2017, a total of 325,000 mobile health (mHealth) apps were available in major app stores, and the number of users of mHealth apps will continue to rise in the upcoming years [1]. These apps have the potential to facilitate tracking of health-related behaviors and weight management [2]. Within this group of apps, diet-tracking apps are very popular, with some downloaded as much as 50 million times (based on MyFitnessPal for Android market, April 2017). Tracking the consumption of certain foods and drinks may potentially help individuals achieve an improved understanding of their dietary patterns [3]. Using a diet-tracking app may improve self-monitoring, goal setting, and knowledge and develop self-efficacy—all of which are key behavior change constructs [2-6]. However, it remains unclear how many of the current diet-tracking apps employ such features that are consistent with behavior change theory. In past reviews of diet-related health apps, it was found that adoption of behavior change theory tended to be quite poor [7-11].

Given the current obesity epidemic within the United States [12] and in many other countries around the world [13], there is a great need for effective theory-driven tools to help individuals manage weight. Aside from tracking energy intake, other aspects of diet may be important to monitor. For instance, shifts in diet toward greater intake of processed foods, meals outside of the home, and greater amounts of oil and sugar-added foods have been implicated as potential causes of the global obesity epidemic [14]. Within the United States, shifts in portion sizes may be responsible for increased energy intake and obesity [15]. Moreover, diet tracking may be particularly useful for those who are at risk of or who already need to manage specific diet-related health issues, such as a need to monitor carbohydrate intake for metabolic disease syndrome [16], sodium intake for hypertension [17], or food elimination for irritable bowel syndrome [18] or allergies [19]. Therefore, the ability for apps to accurately track food intakes may be useful for understanding the changing diet trends within certain populations, as well as diet patterns relevant to an individual’s health. Yet, few studies have been conducted evaluating the accuracy of diet-tracking apps. For example, a single user recording a 3-day diet in a study found that the accuracy in tracking energy intake among apps was only fair in terms of total calories and amounts of macro- and micronutrients compared with a gold standard nutrient coding and that there was large variability among apps [2].

In addition to the importance of accurate diet measures, usability is an important aspect of quality of diet-tracking apps. Usability encompasses multiple dimensions of user interaction with an app, which includes ease of use, complexity, need for training and support, and willingness to continue use. Various weight loss apps have been scored for usability in single-user studies [2], with apps generally receiving good to very good ratings of usability. Moreover, acceptability of mobile apps (ie, the willingness of people to use the apps) for health interventions has generally been found to be high among study participants [10]. However, usability and acceptance may change, particularly as the number of apps focused on diet-tracking increases, and apps become more complex with additional new features.

### Objectives

The goal of our study was to expand upon the existing literature that evaluates health apps in general, with a more focused review of the top Android and iPhone operating system (iOS) diet-tracking apps to evaluate multiple aspects of quality and applicability for use in behavior change studies. This review includes ratings of app usability and coherence of app features with behavior change theory using a 50-item list adapted from the Theoretical Domains Framework (TDF). We further assessed each app’s ability to code calorie, carbohydrate, fat, and protein intake compared with the United States Department of Agriculture (USDA) reference coding.

## Methods

### Selection of Diet-Tracking Apps

Apps were identified using terms *diet tracking* and *diet app* in both Android and iTunes stores in April 2017. iTunes returned the top 100 iPhone apps in both cases but did not display less relevant items. Android Play store returned 245 results. The following categories were used to screen the apps: (1) ability to record dietary intake of users, (2) free of charge, (3) availability for both iOS and Android devices, and (4) high popularity ranking in app store (based on an algorithm that looks at average app store ratings, rating and review volume, download counts, and app usage statistics generated by the App Store and Google Play [20,21]). We selected apps that were free of charge because of their wider user base and number of downloads. We selected the top 7 apps (Table 1). Beyond these 7, the remaining apps were either much less popular with few reviews or appeared in only 1 of the app stores. We refer to some of the apps by their producer names for simplicity in the results below.

**Table 1:** Characteristics of the diet-tracking apps used in the United States.

App name (year established)	Producer (country of origin)	Rating (Android/iOS/ratings)	Downloads (Android)^a^
Calories Counter (2018)	FatSecret (Australia)	4.4/4.5/1300	10,000,000
LifeSum (2018)	LifeSum (Sweden)	4.3/4.5/22,600	5,000,000
MyPlate Calorie Tracker (2017)	Livestrong (United States)	4.6/4.5/7900	500,000
Argus (2015)	Azumio (United States)	4.3/4.5/22,600	100,000
Lose It! (2008)	FitNow (United States)	4.4/4.0/156,600	5,000,000
Calorie Counter & Diet Tracker (2009)	MyFitnessPal (United States)	4.6/4.5/490,000	50,000,000
MyDietCoach (2014)	InspiredApps (United States)	4.4/4.5/5700	10,000,000

^a^No download statistics available for iOS.

### Evaluation Criteria

Usability was scored according to the System Usability Scale (SUS)—a 10-item questionnaire developed by Brooke in 1986 [22], which has been used to evaluate usability for a variety of electronic devices and systems, including health-related smartphone apps [23]. The SUS is a valid and widely used de facto standard for assessing usability [24-26]. As described by Brooke, the SUS aims to assess the degree to which a system is *fit for purpose*. Its questions assess multiple aspects of usability, including ease of use and complexity, and learning and expertise required to use a system. Each question is graded on a 5-point Likert scale ranging from *Strongly agree* to *Strongly disagree*. A composite usability measure is calculated by summing the scores for the odd numbered questions and *5 minus* the score for each of the even numbered questions and multiplying the result by 2.5. The resulting SUS measure ranges from 0 to 100, with a measure above 68 being considered *above average* [27].

Functionality was evaluated by the TDF developed and validated by Cane et al [28]. Developed for behavioral change research, the TDF groups 112 theoretical constructs into 14 domains: Knowledge, Skills, Social/Professional Role and Identity, Beliefs about Capabilities, Optimism, Beliefs about Consequences, Reinforcement, Intentions, Goals, Memory, Attention and Decision Processes, Environmental Context and Resources, Social Influences, Emotions, and Behavioral Regulation, which could be integrated with health behavior change theories, such as the Transtheoretical Model/Stages of Change [29]. The TDF was validated using word sort and clustering exercises by behavior change experts. On the basis of the domains in the TDF, we created a checklist of 50 questions that quantify the presence of diet-tracking app features that relate to specific TDF domains. The questions were developed a priori of using the apps. Each question was designed iteratively by the researchers after reviewing TDF domains and subdomains, discussing the intended meaning of the domain, and how it might manifest as an app feature. Furthermore, the wording of each question was discussed to ensure clarity and ease of scoring. Despite careful wording, because the presence of a feature may not be clear, if reviewers were discordant in their response for a feature, the discordance was discussed, the app was re-reviewed, and consensus was determined by the reviewers.

As all of the apps were targeted for general consumer use and not tied to particular professional services (eg, patient care programs and nutritional services), we focused the Social/Professional Role and Identity domain on characterizing user identity. For example, the question *Does the app make use of Avatars?* relates to a feature that helps the user establish an *Identity* within the app. Similarly, for other domains, we identified specific app features. The question *Does the app provide any encouraging messages?* relates to *Optimism*, and the question *Does the app reward the user in some way (eg, stars, accolades, and achievements) for using of the app?* relates to *reinforcement*. The full list of app functionality questions is in Multimedia Appendix 1.

### App Evaluation

Among the authors of this study, 3 authors who were undergraduate students trained in Nutritional Sciences at the time of the assessment downloaded and evaluated each of the apps for a 2-week period. Using their own smartphones, 2 of the authors used the iOS version of the apps, whereas the third author used the Android version of the apps. After using each app, each researcher independently rated the usability and functionality of the app according to the evaluation criteria. The mean SUS measure was computed for each app, as well as mean scores for individual SUS items. The presence or absence of specific app features (functionality) were noted and compared for each app between the 3 users, and discordances were reviewed and discussed to come to a consensus. Responses on questions related to app features were grouped, and positive responses were summed to create a score. Descriptive statistics for these scores were computed for each domain in the TDF. Correlations of SUS score were calculated among the 3 authors, and mean app SUS was compared with app functionality.

Finally, each user evaluated the nutrient coding of the apps using their 3-day record of all foods consumed using USDA. They recorded all foods consumed in real time for 3 consecutive days that included 2 weekdays and a weekend day (Thursday, Friday, and Saturday) during the 2-week period for each app. At the end of each day, the nutrient intakes (total calories, protein, fat, and carbohydrate) from all food consumed as estimated by each app were noted. The resulting nutrient measures were averaged across the 3 days for each user and each app. The 3-day diet records were also coded by each researcher separately using the USDA Food Composition Database [30]. Portion sizes were estimated based on the researchers’ prior nutritional training. Mean differences between each app’s 3-day average and the USDA reference was computed across the 3 users.

As an additional assessment of coding accuracy, 3 common example food items were input into each app and the USDA database to examine consistency in estimates of calories and macronutrients. A medium banana, a plain Nature Valley granola bar, and a Big Mac from McDonald’s were chosen as examples of a common and popular fruit, a packaged food, and a fast food item, respectively. For the 2 processed foods, food label nutrient data were also recorded.

## Results

### Usability

**Figure 1:**
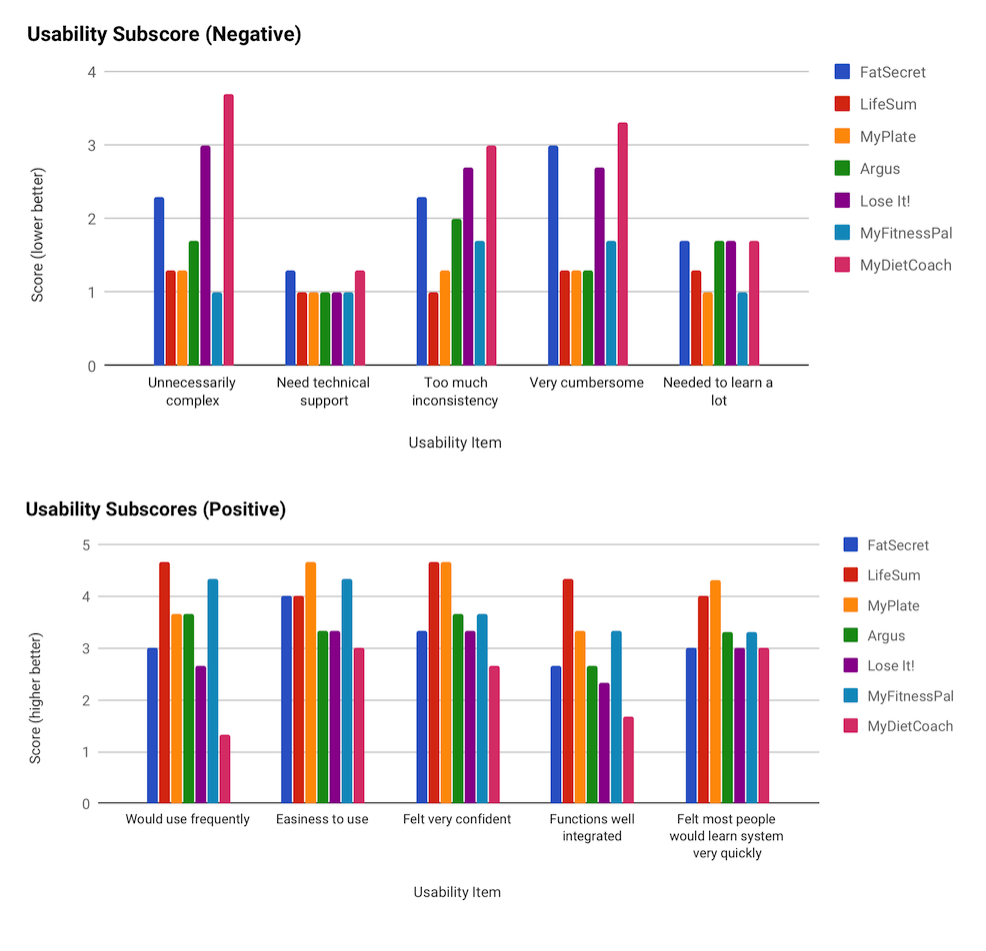
Positive items (top) and negative items (bottom) of usability.

Figure 1 illustrates both the positive and negative aspects of usability from the SUS. Detailed usability subscores (averages of the reviewers) and the aggregate SUS score for each app are reported in Multimedia Appendix 2. The users’ usability scores were consistent and positively correlated with each other and ranged from moderate to high correlation (Pearson correlations of 0.66, 0.84, and 0.89). High contrast scores were not observed. However, there were some notable differences between the iOS and Android versions of the same app; for example, the iOS version of FatSecret had a slightly different user interface and had additional features that made it unnecessarily complex compared with the Android version, which resulted in larger variation in this app’s final SUS score compared with the other apps.

Out of the 7 apps in the study, LifeSum was found to be the most user-friendly app based on the SUS scale with an average SUS score of 89.2, whereas MyDietCoach was found to be the least user-friendly app with an average SUS score of 46.7. Comparing the usability subscores among all 7 apps, LifeSum consistently had high scores among the positive usability subcategories, such as for the following items: *Would use frequently*, *Easiness to use*, and *Felt very confident*. Conversely, MyDietCoach had consistently low usability subscores among these usability subcategories.

For the items that represented aspects of negative usability, such as *Unnecessarily complex*, *Needs technical support*, and *Too much inconsistency*, lower scores in this category indicated that the app was more usable. LifeSum had the lowest scores among almost all of those items, which indicates that the app is more user friendly than the other apps that were tested. MyDietCoach had higher scores among these items, which suggests that the app is less usable than the others. These findings are consistent with the other usability subscores that asked positive usability questions.

Generally, all of the apps scored well in terms of ease of use and did not require considerable amount of learning or technical support. Of particular note, many of the apps have features that greatly improve the ease of entering food items. For instance, 6 out of the 7 apps utilize bar code scanning for input of packaged food, and all of the 7 apps are able to remember recent or frequent food items for quick input. LoseIt! also has a feature that attempts to recognize a food item from a photo.

### Functionality

Each app was evaluated with a 50-question checklist to identify features that could potentially change the users’ behavior based on the TDF. Some apps showed feature discrepancies between the Android and iOS versions. The biggest functionality discrepancy was found in MyDietCoach, whose iOS version allowed diet recording and analysis for free, whereas the Android version required payment to unlock the feature. A feature was counted as present if it was available in at least one of the iOS or Android versions. The complete feature scoring of the apps can be found in Multimedia Appendix 1.

**Figure 2:**
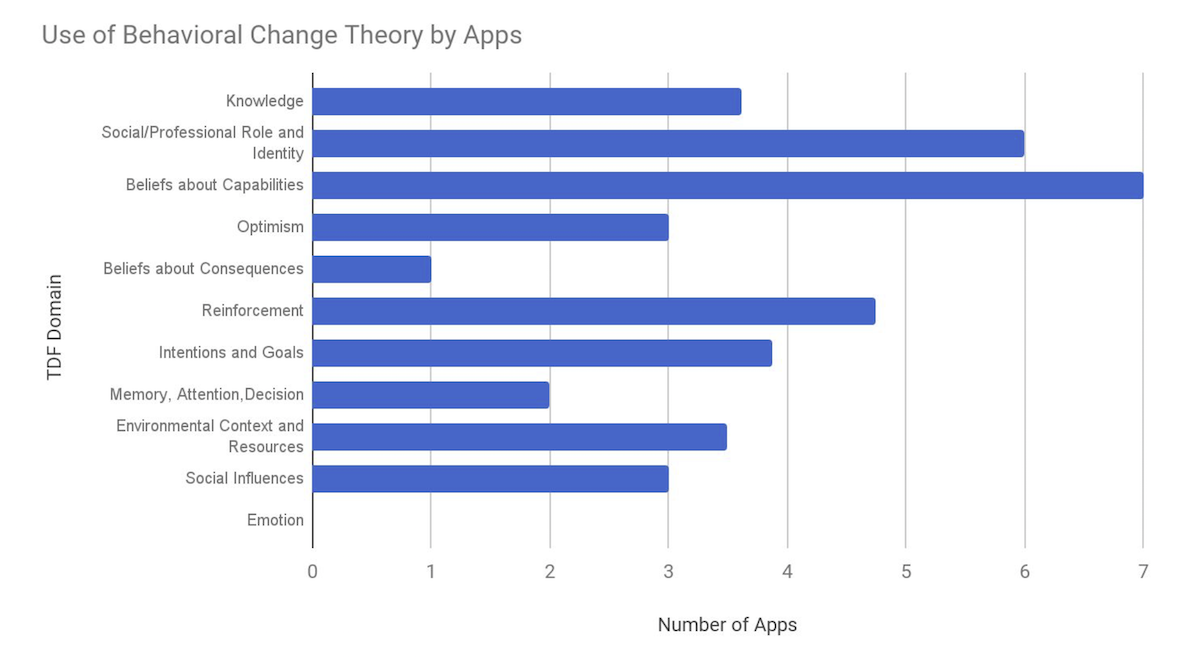
Number of apps that contain specific TDF domain features (for domains with multiple feature checklist questions, the average number of apps across the questions in the domain is reported). TDF: Theoretical Domains Framework.

Figure 2 illustrates the number of apps that had features within a particular TDF domain (for domains with multiple feature checklist questions, the average number of apps across the questions in the domain is reported). Notably, all apps had features for the *Belief about Capabilities* domain, which emphasizes building self-efficacy through tracking of progress and working toward goals. Most of the apps also had features within the *Social/Professional Role and Identity* domain, which tries to tailor to the users’ requirements by establishing a user identity through account registration, use of avatars, and by tracking user-specific profile information.

With respect to the domains that were less featured in the apps, none of the apps had an *Emotion* feature. For example, the apps did not track the effect of diet on mood or stress nor did they try to explicitly track users’ guilt associated with eating certain foods. None of the apps allowed users to note flavor, which is an important aspect of taste, nor did they track users’ hunger or satiety. The next least prominent behavior change domain was the *Beliefs about Consequences* domain, which was only present in 1 app, LifeSum, which had a Health Test feature that assessed and challenged users about their diet beliefs and knowledge.

**Figure 3:**
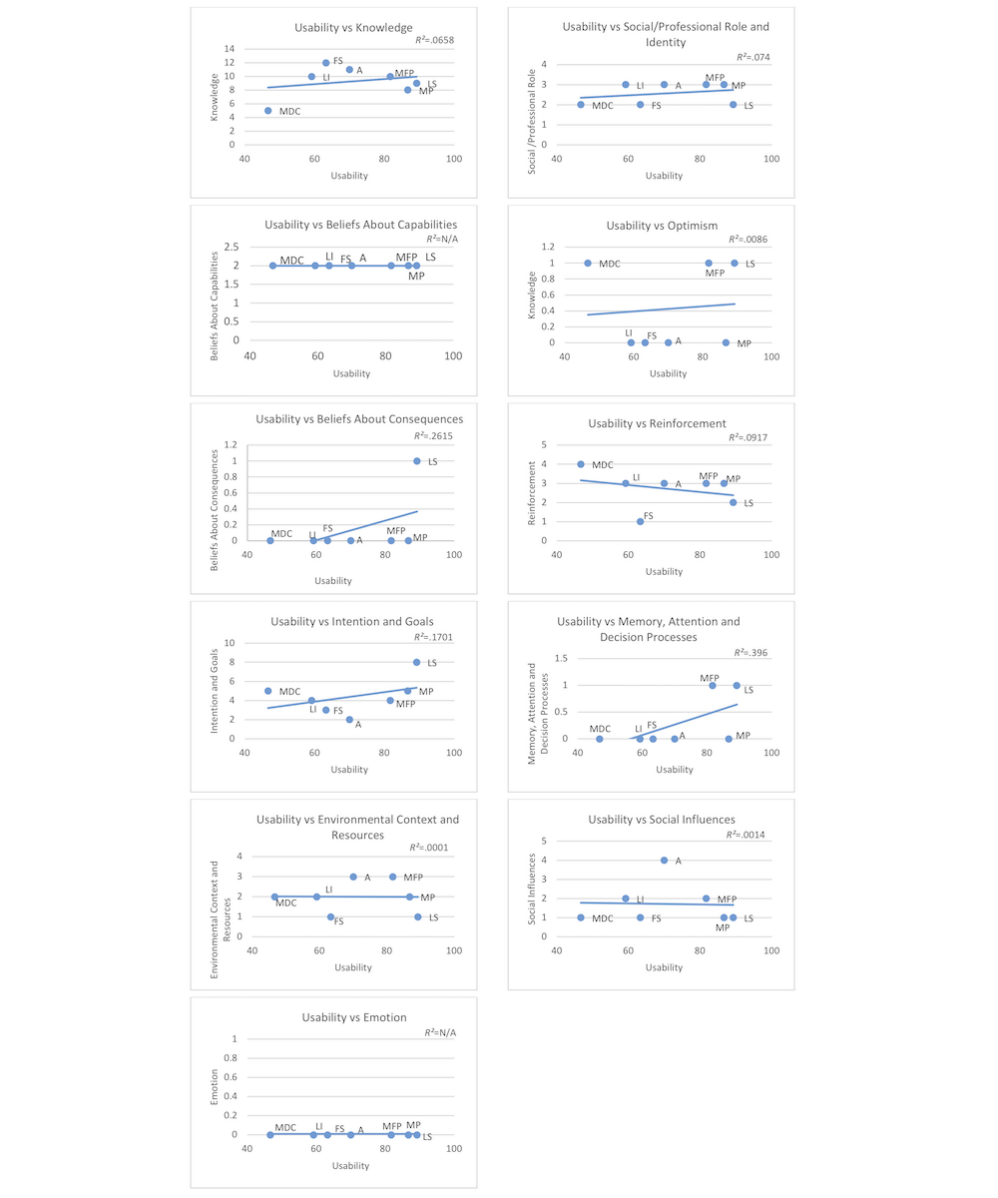
Individual feature correlation with usability. MDC: MyDietCoach; LI: Lose It!; FS: FatSecret; A: Argus; MFP: MyFitnessPal; MP: MyPlate; LS: LifeSum.

Figure 3 illustrates which behavior change domain features are present in each app (linear models are presented in Multimedia Appendix 3). The apps are ordered on the x-axis in increasing SUS score to illustrate the relationship between each feature and overall usability. Generally, there was little correlation between app behavioral domain features and usability (*R*^2^: 0-.396), with *Beliefs about Consequences* and *Memory, Attention, and Decision* being the features most correlated with greater usability (*R*^2^ of .396 and .262, respectively).

*Reinforcement* was the only domain that was negatively correlated with usability, although the correlation was not strong (*R*^2^=.094). Notably 1 of the apps, MyDietCoach, had a number of features that related to reinforcement, including rewarding the user (eg, stars, accolades, and achievements), having game-like functions, and providing occasional reminders. However, the app did not score as well as others in terms of usability, illustrating that feature richness does not necessarily relate to greater usability.

**Figure 4:**
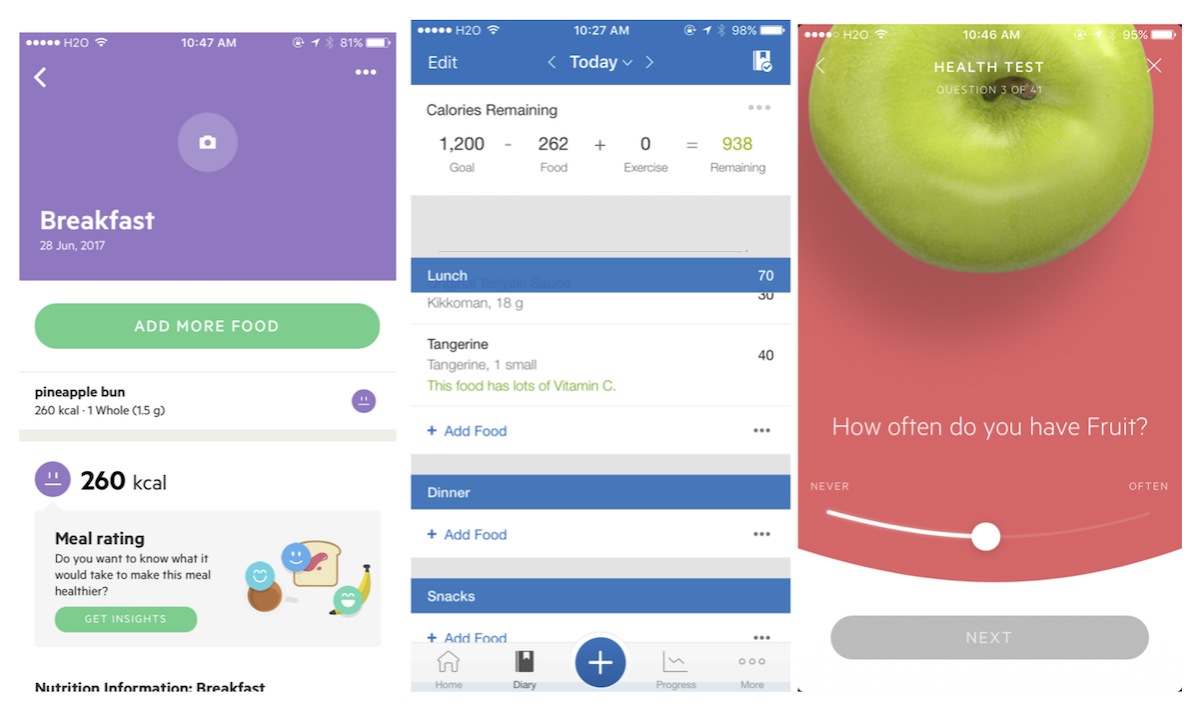
Meal rating feature with emojis in LifeSum (left), feedbacks on nutrient content for food items in MyFitnessPal (center), and Health test in LifeSum (right).

Figure 4 provides example screen captures that illustrate how some of the apps implement features related to behavioral constructs. The 2 apps that ranked high on usability, MyFitnessPal and LifeSum, were the only apps that featured the *Memory, Attention, and Decision* domain. A decision-making feature in an app provides the user with judgments about the quality of their diet choices. Figure 4 shows an example of this judgment from LifeSum, which provides different facial *emoji* icons that are related to the calorie content of food items recorded. Similarly, a screen from MyFitnessPal lets the user know that “this food has lots of vitamin C.” The app with the highest SUS score, LifeSum, was the only app that featured the *Beliefs about Consequences* domain. Figure 4 illustrates how it uses a *Health Test*, which assesses and challenges the user’s belief and knowledge about a healthy diet.

### Consistency Between Apps’ Nutrient Intake Estimates

**Figure 5:**
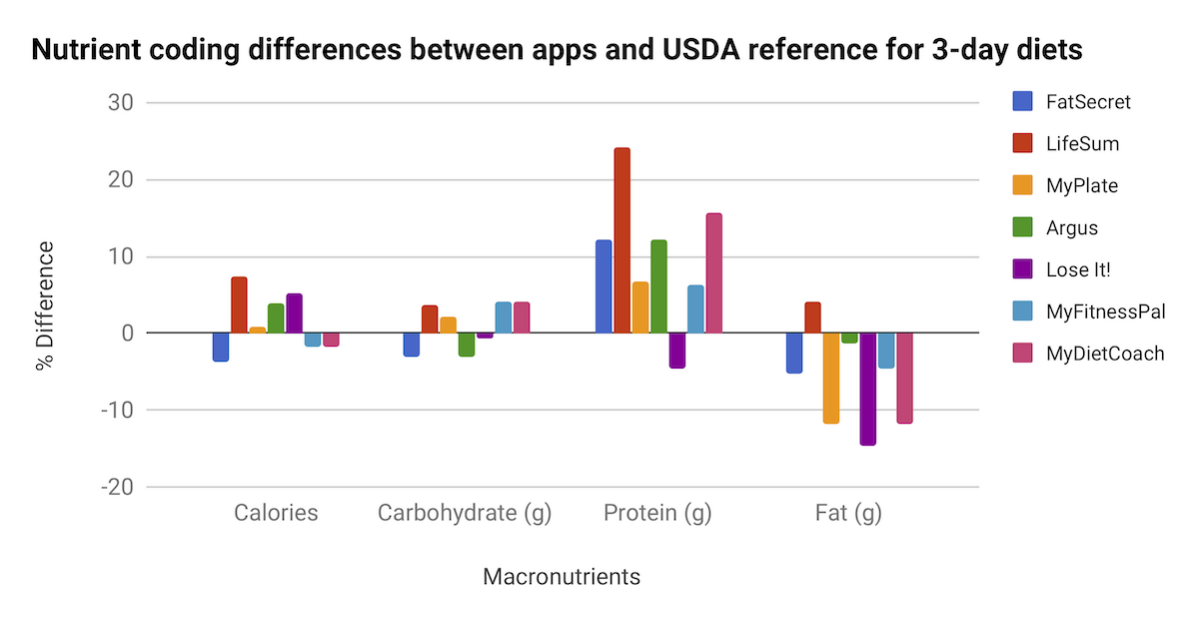
Difference in macronutrient measurements from 3-day diets in 1 week. USDA: United States Department of Agriculture.

On the basis of the subjects’ 3-day intakes, we observed differences in each app’s estimated averages of 3-day total calorie (kcal) and macronutrient intakes (g) compared with calorie and macronutrient estimates generated by using the USDA Food Composition Database (Figure 5). As there was no free diet-tracking feature in the Android version of MyDietCoach, only the 2 iOS users’ data were used for that app. The average total calorie intake was relatively similar among apps, with an average difference of 1.4% compared with the USDA (mean difference 9.6 [SD 50.3] kcal). LifeSum had the highest deviation, an overestimate of 7.29% (SD 70.3) kcal compared with the USDA. On average, the apps only slightly overestimated carbohydrate intake by a difference of 1.0% compared with the USDA (mean difference .8 [SD 4.7] g). With the exception of Lose It!, which slightly underestimated protein intake, most of the apps tended to greatly overestimate intake compared with the USDA (difference of 10.4% and mean difference 3.2 [SD 5.2] g). On average, the apps underestimated fat intake by a difference of 6.5% compared with the USDA. The app that tended to be most accurate in coding calorie and macronutrients relative to the USDA reference was MyFitnessPal, whereas the least accurate was LifeSum.

As an additional assessment of coding accuracy, a ripe medium banana, a plain Nature Valley granola bar, and a Big Mac from McDonald’s were coded using each app and the USDA database (Table 2). We found high consistency for caloric content of a medium banana across the apps and the USDA database with average difference of 3.7 kcal (3.5%) compared with USDA. All apps showed consistent estimates except for LifeSum. LifeSum showed higher calories and macronutrient. This may be due to portion size variations, as the only banana item listed in that app was for a weight of 130 g, which may be larger than a *medium banana* portion size provided by the other apps. A large difference was found for the Nature Valley granola bar, with average caloric difference of −8.4 kcal (−4.2%). Interestingly, most apps showed better consistency with the bar’s food label (190 kcal) rather than the USDA database (203 kcal). The food label indicated not only slightly lower calories but also lower protein (3 g vs 4 g) and fat content (7 g vs 9 g) compared with the USDA database. For the granola bar, again LifeSum showed the greatest discrepancy in calories compared with USDA. McDonald’s Big Mac had the largest difference among the 3 food items, with average difference of −7.9% in calories. All the apps tended to underestimate calories and macronutrients compared with the USDA. Nutrient data found on McDonald’s website also had lower values for this food item than USDA’s data. In total, 2 of the apps were consistent with the nutrition data provided by McDonald’s, whereas the others were not.

**Table 2:** Example of nutrient coding differences between apps for 3 food items.

Food item		FatSecret	LifeSum	MyPlate	Argus	Lose It!	MyFitnessPal	MyDietCoach	United States Department of Agriculture	Food label
**Banana (1 medium, ripe)^a^**										
	Calories (kcal)	105	131	105	105	105	105	105	105	—^b^
	Carbohydrate (g)	26.95	29.7	26.95	27	27	27	26.95	27	—
	Protein (g)	1.29	1.4	1.29	1.3	1.3	1.3	1.29	1	—
	Fat (g)	0.39	0.7	0.39	0.4	0.4	0.4	0.39	0	—
**Nature Valley (plain, crunchy, 1 package/2 bar)^c^**										
	Calories (kcal)	190	220	190	192	190	190	190	203	190
	Carbohydrate (g)	29	29	29	27.1	29	29	29	28	29
	Protein (g)	4	4	4	3.4	3	4	4	4	3
	Fat (g)	6	11	6	7.2	7	6	6	9	7
**McDonald’s Big Mac (1 sandwich)^d^**										
	Calories (kcal)	530	518	540	563	540	550	530	585	540
	Carbohydrate (g)	47	45	47	44	46	46	47	48	47
	Protein (g)	24	24	25	25.9	25	25	24	27	25
	Fat (g)	27	29	28	32.8	28	29	27	32	28

^a^LifeSum only had 1 banana (130 g) food item; Argus and MyFitnessPal had multiple verified banana items.

^b^Not applicable.

^c^LifeSum had multiple food items for *Nature valley crunchy*; the first was used.

^d^MyPlate and Argus contained multiple food items for Big Mac; the first was used. MyFitnessPal had multiple food items for Big Mac; the fourth one with the verified restaurant label that was not the United Kingdom or Canadian version was used.

## Discussion

### Principal Findings

Our review found that current diet-tracking apps generally scored well in terms of usability with 4 apps having SUS scores of 70 or above. Usability may affect compliance and the willingness of users to use these apps in behavior change studies [2]. In this study, for the 3-day diet record, we entered food items into the apps as well as the USDA website and found that the apps were easier to use compared with traditional diet coding approaches. Notably, the apps make use of features such as barcode scanning, photo entry, lists of frequently entered food items, and *auto-completion* —a short list of suggestions of food items by entering only a few letters of a food item word, which greatly increase the ease in which items can be entered. The databases in some of the apps may provide more convenient nutrient coding. For example, 1 of our coders tried entering a *tofu rice bowl* —an integrated food item made up of multiple components. For the USDA database, coders would need to estimate the portion of, and input each individual component, including condiments into the database, whereas the single *tofu rice bowl* item was already in the app’s database. If users take the time to enter each component of a composite food item, and do so completely, energy and nutrient composition may be more accurately assessed than simply selecting a generic composite food item because there may be large variations in how different restaurants make the dish.

In evaluating apps, we noticed some inconsistencies between iOS and Android versions of the same app, which can affect both usability and features. These differences may exist because the iOS and Android platforms and their underlying user interfaces are inherently different. The operating systems also have different feature sets and application programming interfaces. Furthermore, the apps are typically coded in different programming languages (Java for Android and Objective C for iOS). Although there has been progress in the development of application frameworks, such as those that leverage HTML5, that allow for cross-platform app development, not all the diet-tracking apps may rely on these frameworks or the frameworks may still allow for platform-specific design choices that affect use. Due to the potential difference in iOS versus Android versions, researchers should carefully evaluate both versions of a diet-tracking app to ensure that they have similar features before using the app for a behavior change study.

Our findings differ slightly from the previous diet-related app review study [11], which found that the apps generally lacked adherence to behavior change theory. In our study, we found that some elements of behavior change theory are beginning to be implemented in some of the most popular diet-tracking apps. For instance, all of the apps we reviewed promote self-efficacy by allowing users to track their diet progress and work toward simple personal goals. Described by Bandura in 1977 [31], self-efficacy is a key behavioral theory that describes a person’s belief and expectations that they can accomplish certain tasks, which could include meeting dietary goals (eg, weight loss, balanced diets, and following certain diet patterns). However, despite the presence of features in apps that could potentially be used to improve self-efficacy, the evidence regarding the effectiveness for diet-tracking apps is still limited. Future studies, particularly those conducted in real-world contexts will need to evaluate whether users’ self-efficacy in meeting their dietary goals is enhanced by using certain apps.

The presence of behavior change features generally tended to be correlated with higher usability. This is encouraging, as apps may be selected by researchers for their studies because they adhere to known and specific behavior change constructs. At the same time, having specific behavior change functions does not necessarily make the apps less enjoyable to use for study participants. However, not all the features were positively correlated with usability. Notably, the *Reinforcement* domain, which in our case, related to user rewards such as giving stars, accolades, achievements, etc, was slightly negatively associated with usability. Although gamification is generally thought of as a powerful behavior change strategy used within technology [32], it may be that game-like features within diet-tracking apps may detract from the core functionality of recording diet and observing nutrition estimates. Further research may be needed to explore the role of reinforcement from diet-tracking apps in intervention studies.

The goal of this study was not to evaluate the effectiveness of specific behavior change features in altering diet; however, we did observe considerable variations between the diet-tracking apps, which make selecting particular apps for intervention studies an important consideration. No app is inherently better than another in terms of features, but instead, certain apps might be more appropriate for use within particular studies because they are stronger in specific behavior change domains than others and better fit an overall intervention strategy. Behaviorists may choose certain apps because they reinforce the domains of focus for their health education and other interactions with subjects. In doing so, having their subjects use an appropriate app could be a complement to in-person work. The app may bridge the gaps between in-person visits, allowing the subjects to explore their own diet activity [8,33,34]. Despite these opportunities, a survey conducted of the Australian, New Zealand, and British dietetic association members (ie, registered dietitians and practitioners) found that although diet app usage is high, the apps have yet to be fully integrated into nutritional care practice or within behavior change programs [35]. Figure 3 and the detailed feature checklist results in Multimedia Appendix 1 may help behaviorists identify appropriate apps. The American Academy of Nutrition and Dietetics regularly reviews individual apps, which may be an additional resource [36].

Although it is important to acknowledge variations between the apps, the presence and absence of features within certain apps are noteworthy. For instance, LifeSum scored the highest in usability and had a unique feature—a Health Test. We did not evaluate the accuracy of their test, but the concept of integrating routine knowledge/belief assessments, while tracking its effect on changes in the users’ diet tracking, has the potential to be a useful intervention tool. We also noticed that features related to emotions were missing from all the apps despite considerable research, which has identified the associations between both positive and negative emotions and diet [37-41]. Moreover, tracking of potential upstream determinants and downstream effects of diet (eg, hunger, satiety, guilt, stress, happiness, and taste) tend be to lacking from these diet-tracking apps but would potentially be useful to track to assess associations with diet patterns. These missing behavioral domains could be an important future area for diet-tracking app development.

As part of our testing, we evaluated the accuracy of a 3-day diet record compared with the USDA reference. Generally, we observed that tracking of calories and carbohydrates closely matched estimates from the USDA database; however, there were large inconsistencies between apps with respect to protein and fat estimates. This is somewhat surprising as proteins and fats are both components of caloric intake. We suspect the underestimation of fat may be due to the difficulties in estimating oils used in cooking, particularly for restaurant meals. These differences could have implications for those with specific health issues. For example, for individuals with cardiovascular disease who rely on such apps to track their fat intake, underestimation of their fat intake could contribute to high blood cholesterol levels and exacerbate their health conditions. This led us to take a closer look at select food items to better understand how the apps may vary from the USDA reference. Notably 2 of our items, the granola bar and fast food sandwich, have food labels, and we found that the USDA nutritional values differed from the labeling. In some cases, we observed that although the apps were not consistent with USDA, they were consistent with the food label. We found the unlabeled item, the banana, to be more consistent with USDA than the other 2 items. Perhaps food reformulations may make some databases, including USDA’s, more easily outdated compared with unprocessed food items. Furthermore, because some of the apps rely upon user suggestions for nutrient content, it may be that more users submit requests for updated caloric content for certain macronutrients for food items.

Interestingly, the app with highest usability, LifeSum, had the greatest nutrient coding inconsistency compared with the USDA reference. This presents a potential challenge for interventions, which rely upon accurate estimates from apps, instead of having trained staff recode the food items using a standard reference database. We conducted additional research to learn about the database used by LifeSum and found that it uses a series of databases, which includes the USDA database, as well as MyNetDiary, UK Food Standards Agency (United Kingdom), Bundeslebensmittelschlüssel (Germany), Livsmedelverket (Sweden), and food contributed by users (new food items created from users by entering nutrient details) [42]. LifeSum’s headquarters is located in Sweden, and perhaps because of this, it may be more accommodating of European users. The app’s nutrient coding may be more accurate when compared with the European databases, although we did not perform this evaluation. As another example, we researched the development of MyFitnessPal (the app we found to be most consistent with USDA’s database). The app initially started with its creator entering food items, and later, relied upon user crowdsourcing (ie, users entering the nutritional content of food items into their database) [43]. Consistent with other studies, despite underestimation of nutrients, MyFitnessPal had the highest correlation with the USDA database [44-46]. Of note, the study by Chen et al is 1 of the only studies in which participants recruited from the general population of smartphone users in Australia, who had not used an app (MyFitnessPal), were asked to use the app to record their dietary intake in a close to real-world context (ie, they were asked to install and use the app, were recruited specifically for the study, which also included phone-based 24-hour recalls) and found that daily energy intake was significantly underestimated by 445 kcal [47], whereas studies similar to ours [45], which rely upon users with nutritional training to use the app are likely to observe more complete and accurate results. Teixeira et al [46] recruited non-nutrition major university students in a study comparing paper versus app (MyFitnessPal) in Brazil and found that while many nutrients were underestimated compared with paper records, there was moderate correlation between both methods. Griffiths et al [45] who relied upon research staff to dietary recall data into different tracking apps also observed significant differences in app-reported nutrient levels compared with the standard they used for their research (the Nutrition Data System for Research). Due to these inconsistencies, Chen et al [47] recommended the use of apps with guidance from dietitians if more accurate dietary data are required.

With the inclusion of elements of behavior change theory into popular diet-tracking apps, there may be reasons to incorporate apps into interventions that leverage these features. However, there could also be disadvantages of using smartphone diet/nutrition apps. These include increased screen time, usability and acceptance issues, as well as concerns about users’ privacy [48]. In addition, there is some emerging evidence that suggests that some diet-tracking apps may be frequently used by those with eating disorders, and their use may be perceived as contributing to their disorder [49]. For individuals with eating disorders, caution need to be exercised for the use of diet/nutrition apps, which may not be appropriate replacements for clinical treatment or medical monitoring [48,50].

### Limitations

There are potential limitations of our research that are worth noting. First, the apps were reviewed by 3 college-aged authors who are technology-savvy and trained in nutritional science. This was necessary to conduct an accurate review of the apps’ features and accurate diet coding. However, their usability scores may not generalize to populations who do not have training in nutrition. Due to digital entitlement [51], certain populations do not have access to smart devices, and it cannot be assumed that all users will be similarly comfortable with using these apps. We did not conduct any inter- or intrarater reliability assessment for the 3 reviewers and the measurement tools used. Future studies should include both inter- and intrarater reliability assessments and more users.

Of the 7 apps we evaluated, 5 originated from the United States. These apps may be using US food databases only, which may not be applicable in other countries because of the differences in food processing, regulations, and policies.

There may also be issues generalizing some of the 3-day diet findings to what might occur in a more general population. We conducted diet assessment of 3 consecutive days because of its relative ease and less reviewer burden. Consecutive days may limit the variation in the food intake compared with nonconsecutive days; however, we did include 2 weekdays and 1 weekend day to capture more variety in the authors’ food intake. The authors’ diet coding may be more accurate than what users in general may be able to achieve, as the authors have a better idea of the caloric content of certain foods, allowing them to quickly catch coding errors. In addition, they may be able to estimate serving sizes better compared with normal users. Another potential issue that may affect broader generalization is that the average caloric intake of the 3 authors during the 3-day period was lower than the average caloric intake for US adults, which is 2091 kcal per day for 2007-2010 [30]. If the caloric intake was higher on the 3 days, the discrepancies between the apps and the USDA reference may potentially have been higher. Furthermore, energy underreporting is common with diet assessment, which may compound the problem of underestimation of food intake, particularly with apps as has been found by others [47]. However, other researchers have noted that although self-reported energy intake may not accurately reflect true energy intake, self-report methods can still provide valuable information about foods and beverages consumed by populations, which can be used to inform nutritional policy and associations between diet and disease [52]. The origin and types of food items (Mexican banana vs banana from other origins) may contribute to their nutrient and caloric variabilities. However, we did not include this information in our nutrient coding.

This is the first use of a behavior change checklist to assess diet-tracking apps, and although questions were developed through an iterative process to align them with a behavior change framework, the checklist has not been thoroughly validated. Despite these limitations, our review provides a broad assessment of the potential use of the current generation of diet-tracking apps for diet intervention studies. Given the popularity of these apps, further research to evaluate the effectiveness of interventions that use these apps is warranted.

### Conclusions

This study showed that the 7 most popular diet-tracking apps on Apple App Store and Google Play are feature-rich and easy to use. The apps incorporate features consistent with many behavior change domains, notably promoting self-efficacy through tracking diet and progress toward goals. Although the relatively large deviations in coding of protein and fat compared with the USDA reference deserve further examination, the apps performed similarly to the USDA in coding calories and carbohydrates. Together, these aspects allow these diet-tracking apps to be useful for a wide range of dietary intervention studies.

## Appendix

Multimedia Appendix 1 App feature checklist and corresponding Theoretical Domains Framework (TDF) domain and construct (discordant indicates differences between iOS and Android versions).

Multimedia Appendix 2 System Usability Scale scores.

Multimedia Appendix 3 Correlation coefficients, standard errors, and P values, and R2 for linear models of usability versus TDF domain features.

## Abbreviations

iOS: iPhone operating system
mHealth: mobile health
SUS: System Usability Scale
TDF: Theoretical Domains Framework
USDA: United States Department of Agriculture
