# Supplementary material for: A Focused Review of Smartphone Diet-Tracking Apps: Usability, Functionality, Coherence With Behavior Change Theory, and Comparative Validity of Nutrient Intake and Energy Estimates
Source: JMIR Mhealth Uhealth. 2019 May 17;7(5):e9232. doi: 10.2196/mhealth.9232 (PMC6543803; doi:10.2196/mhealth.9232)
Supplement: Multimedia Appendix 1 [file mhealth_v7i5e9232_app1.docx]

Multimedia Appendix 1

App feature checklist and corresponding Theoretical Domains Framework (TDF) domain and construct (discordant indicates differences between iOS and Android versions).

| **Domain** | **Subdomain** | **Questions** | **FatSecret** | **LifeSum** | **Myplate** | **Argus** | **MyDietCoach** | **Lose It!** | **MyFitnessPal** | **# of apps** |
| --- | --- | --- | --- | --- | --- | --- | --- | --- | --- | --- |
| Knowledge | Establishes diet knowledge | Tracks meal events (e.g., when meals occur)? | yes | discordant | yes | yes | discordant | yes | yes | 7 |
| Knowledge | Establishes diet knowledge | Tracks specific food items? | yes | yes | yes | yes | discordant | yes | yes | 7 |
| Knowledge | Establishes diet knowledge | Tracks portion sizes? | yes | yes | yes | yes | discordant | yes | yes | 7 |
| Knowledge | Establishes diet knowledge | Tracks macronutrients? | yes | yes | yes | yes | no | yes | yes | 6 |
| Knowledge | Establishes diet knowledge | Tracks micronutrients? | yes | discordant | yes | yes | no | no | yes | 5 |
| Knowledge | Establishes diet knowledge | Tracks photos? | yes | no | no | yes | discordant | yes | yes | 5 |
| Knowledge | Establishes diet knowledge | Tracks who they eat with? | no | no | no | yes | no | no | no | 1 |
| Knowledge | Establishes diet knowledge | Tracks where they eat? | no | no | no | yes | no | no | no | 1 |
| Knowledge | Establishes diet knowledge | Does app use any sensors on the phone? | yes | yes | yes | yes | yes | yes | yes | 7 |
| Knowledge | Establishes diet knowledge | Offers alternative food options? | no | no | no | no | no | no | no | 0 |
| Knowledge | Establishes diet knowledge | Provides a nutritional report? | yes | yes | yes | yes | no | yes | yes | 6 |
| Knowledge | Establishes diet knowledge | Does the app provide features specific to cooking/preparing meals (e.g., recipes)? | yes | yes | no | no | no | no | no | 2 |
| Knowledge | Establishes diet knowledge | Does the app provide features specific to eating out? | discordant | no | no | no | no | yes | yes | 3 |
| Knowledge | Establishes diet knowledge | Does the app provide features specific to shopping for groceries or other food items? | discordant | no | no | no | no | yes | no | 2 |
| Knowledge | Establishes diet knowledge | Does the app provide features specific to food deliveries? | no | no | no | no | no | no | no | 0 |
| Knowledge | Establishes diet knowledge | Does the app provide any specific features to categorize different ethnic foods? | no | no | no | no | no | no | no | 0 |
| Knowledge | Establishes diet knowledge | Does the app allow input of food prices? | no | no | no | no | no | no | no | 0 |
| Knowledge | Establishes diet knowledge | Does the app track diet in terms of RDAs | yes | yes | yes | yes | no | yes | yes | 6 |
|  |  |  |  |  |  |  |  |  |  |  |
| Skills |  | (Covered by Usability questions) |  |  |  |  |  |  |  |  |
|  |  |  |  |  |  |  |  |  |  |  |
| Social/Professional Role and Identity | Identity | Does the app require registration to use? | yes | no | yes | yes | no | yes | yes | 5 |
| Social/Professional Role and Identity | Identity | Does the app ask for any user-specific profile info (e.g., gender, age, race/ethnicity, income, location)? | yes | yes | yes | yes | yes | yes | yes | 7 |
| Social/Professional Role and Identity | Identity | Does the app make use of Avatars? | no | yes | yes | yes | yes | yes | yes | 6 |
|  |  |  |  |  |  |  |  |  |  |  |
| Beliefs about Capabilities | Self-efficacy | Does the app allow the user to track progress generally? | yes | yes | yes | yes | discordant | yes | yes | 7 |
| Beliefs about Capabilities | Self-efficacy | Does the app allow the user to track progress toward specific goals or intents? | yes | yes | yes | yes | yes | yes | yes | 7 |
|  |  |  |  |  |  |  |  |  |  |  |
| Optimism | Optimism | Does the app provide any encouraging messages? | no | yes | no | no | yes | no | yes | 3 |
|  |  |  |  |  |  |  |  |  |  |  |
| Beliefs about Consequences | Beliefs | Does the app challenge your diet beliefs (like questions that test knowledge)? | no | yes | no | no | no | no | no | 1 |
|  |  |  |  |  |  |  |  |  |  |  |
| Reinforcement | Rewards | Does the app reward the user in some way (e.g., stars, accolades, achievements) for using of the app? | no | no | discordant | yes | yes | yes | yes | 5 |
| Reinforcement | Incentives | Does the app have game-like features? | no | no | no | no | yes | no | no | 1 |
| Reinforcement | Reinforcement | Does the app provide occasional reminders, notifications, banners, etc? | no | yes | yes | yes | yes | yes | yes | 6 |
| Reinforcement | Consequents | Does the app allow the user to observe potential cause and effect relationships (e.g., diet vs weight)? | yes | yes | yes | yes | discordant | yes | yes | 7 |
|  |  |  |  |  |  |  |  |  |  |  |
| Intentions and Goals | Goals | Does the app ask the user to set goals? | yes | yes | yes | yes | discordant | yes | yes | 7 |
| Intentions and Goals | Goals | Does the app suggest goals? | no | yes | discordant | yes | yes | no | no | 4 |
| Intentions and Goals | Intentions | Does the app ask about intentions? | yes | yes | discordant | no | yes | yes | yes | 6 |
| Intentions and Goals | Intentions | Does the app provide features specific to individuals with cardiovascular risk? | no | discordant | no | no | no | no | no | 1 |
| Intentions and Goals | Intentions | Does the app provide features specific to individuals with diabetes risk? | no | discordant | no | no | no | no | no | 1 |
| Intentions and Goals | Intentions | Does the app provide features specific to individuals with eating disorders? | no | discordant | no | no | no | no | no | 1 |
| Intentions and Goals | Intentions | Does the app provide features specific to weight loss/gain? | yes | yes | yes | no | yes | yes | yes | 6 |
| Intentions and Goals | Action planning | Does the app have the user follow a plan? | no | yes | yes | no | discordant | yes | yes | 5 |
|  |  |  |  |  |  |  |  |  |  |  |
| Memory, Attention and Decision Processes | Decision Making | Does the app question/judge your diet choices (i.e., it's trying to get the user to think about their choices)? | no | yes | no | no | no | no | yes | 2 |
|  |  |  |  |  |  |  |  |  |  |  |
| Environmental Context and Resources | Context | Does the app track exercise? | yes | yes | yes | yes | discordant | yes | yes | 7 |
| Environmental Context and Resources | Context | Does the app allow the user to engage with neighborhood and/or community resources? | no | no | no | no | no | no | no | 0 |
| Environmental Context and Resources | Context | Does the app show ads? | no | no | discordant | discordant | discordant | yes | yes | 5 |
| Environmental Context and Resources | Context | Does the track location (like GPS)? | no | no | no | yes | no | no | yes | 2 |
|  |  |  |  |  |  |  |  |  |  |  |
| Social Influences | Social | Does the app link to social media accounts/apps? | no | yes | no | discordant | no | no | no | 2 |
| Social Influences | Social | Does the app have some sort of community of users? | yes | no | yes | yes | no | yes | yes | 5 |
| Social Influences | Social | Does the app allow for chat? | no | no | no | yes | no | yes | yes | 3 |
| Social Influences | Social | Does the app provide an opportunity for the user to follow a "model" (e.g., a norm, avatar, peer, etc.)? | no | no | no | yes | discordant | no | no | 2 |
|  |  |  |  |  |  |  |  |  |  |  |
| Emotion | Emotion | Does the app provide features for tracking affective state (e.g., mood, stress, guilt) | no | no | no | no | no | no | no | 0 |
| Emotion | Emotion | Does the app allow the user to comment on their appetite or taste for foods (e.g., rate flavor)? | no | no | no | no | no | no | no | 0 |
| Emotion | Emotion | Does the app allow the user to comment on their hunger or satiety? | no | no | no | no | no | no | no | 0 |
