# Supplementary material for: A Focused Review of Smartphone Diet-Tracking Apps: Usability, Functionality, Coherence With Behavior Change Theory, and Comparative Validity of Nutrient Intake and Energy Estimates
Source: JMIR Mhealth Uhealth. 2019 May 17;7(5):e9232. doi: 10.2196/mhealth.9232 (PMC6543803; doi:10.2196/mhealth.9232)
Supplement: Multimedia Appendix 2 [file mhealth_v7i5e9232_app2.docx]

Multimedia Appendix 2

System of Usability Scale Scores

|  | FatSecret^a^ | LifeSum | MyPlate | Argus | Lose It! | MyFitnessPal | MyDietCoach^a^ |
| --- | --- | --- | --- | --- | --- | --- | --- |
| Usability Item | Mean (SD) | Mean (SD) | Mean (SD) | Mean (SD) | Mean (SD) | Mean (SD) | Mean (SD) |
| **Positive Questions** | | | | | | | |
| Would use frequently | 3.0 (1.0) | 4.7 (.6) | 3.7 (.6) | 3.7 (.6) | 2.7 (.6) | 4.3 (.6) | 1.3 (.6) |
| Felt very confident | 3.3 (1.5) | 4.7 (.6) | 4.7 (.6) | 3.7 (.6) | 3.3 (1.2) | 3.7 (.6) | 2.7 (.6) |
| Easiness to use | 4.0 (0) | 4.0 (0) | 4.7 (.6) | 3.3 (.6) | 3.3 (1.2) | 4.3 (.6) | 3.0 (1.0) |
| Functions well integrated | 2.7 (1.5) | 4.3 (.6) | 3.3 (1.2) | 2.7 (.6) | 2.3 (.6) | 3.3 (.6) | 1.7 (.6) |
| Easiness to learn | 3.0 (1.0) | 4.0 (0) | 4.3 (.6) | 3.3 (.6) | 3.0 (1) | 3.3 (.6) | 3.0 (1.0) |
| **Negative Questions** | | | | | | | |
| Very cumbersome | 3.0 (1.0) | 1.3 (.6) | 1.3 (.6) | 1.3 (.6) | 2.7 (.6) | 1.7 (.6) | 3.3 (1.5) |
| Unnecessarily complex | 2.3 (1.5) | 1.3 (.6) | 1.3 (.6) | 2.7 (.6) | 3.0 (1) | 1.0 (0) | 3.7 (1.2) |
| Needed to learn a lot | 1.7 (.6) | 1.3 (.6) | 1.0 (0) | 1.7 (1.2) | 1.7 (1.2) | 1.0 (0) | 1.7 (.6) |
| Need technical support | 1.3 (.6) | 1.0 (0) | 1.0 (0) | 1.0 (0) | 1.0 (0) | 1.0 (0) | 1.3 (.6) |
| Too much inconsistency | 2.3 (1.5) | 1.0 (0) | 1.3 (.6) | 2.0 (0) | 2.7 (.6) | 1.7 (.6) | 3.0 (0) |
| **SUS Score** | **63.3 (18.8)** | **89.2 (1.4)** | **86.7 (3.8)** | **70.0 (5.0)** | **59.2 (12.3)** | **81.7 (3.8)** | **46.7 (12.3)** |

^a^ Large difference between Android and iPhone version of the app
