# Supplementary material for: A Focused Review of Smartphone Diet-Tracking Apps: Usability, Functionality, Coherence With Behavior Change Theory, and Comparative Validity of Nutrient Intake and Energy Estimates
Source: JMIR Mhealth Uhealth. 2019 May 17;7(5):e9232. doi: 10.2196/mhealth.9232 (PMC6543803; doi:10.2196/mhealth.9232)
Supplement: Multimedia Appendix 3 [file mhealth_v7i5e9232_app3.docx]

Multimedia Appendix 3

Correlation coefficients, standard errors, and *P* values, and *R^2^* for linear models of usability vs TDF domain features

|  | β | SE | p | R^2^ |
| --- | --- | --- | --- | --- |
| Knowledge | .037 | .063 | .579 | .066 |
| Social/Professional Role and Identity | .009 | .015 | .555 | .074 |
| Beliefs about Capabilities* | - | - | - | - |
| Optimism | .003 | .015 | .843 | .009 |
| Beliefs about Consequences | .012 | .009 | .241 | .262 |
| Reinforcement | -.018 | .026 | .509 | .092 |
| Intentions and Goals | .050 | .049 | .358 | .170 |
| Memory, Attention and Decision Processes | .020 | .011 | .130 | .396 |
| Environmental Context and Resources | -.001 | .023 | .982 | <.001 |
| Social Influences | -.003 | .032 | .936 | .001 |
| Emotion* | - | - | - | - |

* Unable to fit model due to no variation between apps.
